# Bringing the Next Generation of Immuno-Oncology Biomarkers to the Clinic

**DOI:** 10.3390/biomedicines6010014

**Published:** 2018-02-02

**Authors:** Alessandra Cesano, Sarah Warren

**Affiliations:** NanoString, Inc., Seattle, WA 98109, USA; swarren@nanostring.com

**Keywords:** biomarker, immunotherapy, immuno-oncology, PanCancer IO360, gene expression profiling, gene expression signature, NanoString

## Abstract

The recent successes in the use of immunotherapy to treat cancer have led to a multiplicity of new compounds in development. Novel clinical-grade biomarkers are needed to guide the choice of these agents to obtain the maximal likelihood of patient benefit. Predictive biomarkers for immunotherapy differ from the traditional biomarkers used for targeted therapies: the complexity of the immune response and tumour biology requires a more holistic approach than the use of a single analyte biomarker. This paper reviews novel biomarker approaches for the effective development of immune-oncology therapies, highlighting the promise of the advances in next-generation gene expression profiling that allow biologic information to be efficiently organized and interpreted for a maximum predictive value at the individual patient level.

## 1. Introduction

Immunotherapy is proving to be an effective therapeutic approach in a variety of advanced and metastatic cancers [1]. However, despite the clinical success of the first wave of antibodies against the immune regulator cytotoxic T-lymphocyte-associated protein 4 (CTLA4) and the programmed death-ligand 1 (PD-L1) and programmed cell death protein 1 (PD-1), only a subset of unselected patients exhibits durable responses [2]. Furthermore, the field is witnessing notable failures in Phase 3 trials when these drugs are tested as single agents in early lines of therapy in unselected or sub-optimally selected patient populations [3,4,5,6,7]. Finally, preliminary data indicate that the combinations of these agents, although promising in certain settings, are associated with increased toxicity and cost [8,9]. The number of immune-oncology targets is high and growing and the number of potential combinations of therapeutic agents directed against these targets and of combinations of such agents with conventional standard-of-care agents is even greater. Therefore, the progress in fully realizing the potential of this anticancer approach requires the development and implementation of novel clinical-grade biomarkers able to guide the selection of agents with complementary mechanisms of action targeting multiple mechanisms of resistance and immune escape. This paper will discuss novel biomarker approaches aimed at informing an effective drug development from a mechanistic point of view, as well as the clinical implementation (i.e., patient enrichment) of immune therapies.

## 2. Required Features of an Ideal Immune-Oncology (IO) Patient-Enrichment-Selection (i.e., “Predictive”) Biomarker

Biomarkers in oncology can be broadly categorized as prognostic and predictive. Prognostic biomarkers are defined as biomarkers used to identify the likelihood of a clinical event as part of the natural history of the disease, such as disease recurrence or progression in patients who have the disease or medical condition of interest [10]. These biomarkers are useful for informing patients about the risk of recurrence or the median survival and can also be used for a prospective stratification of the clinical trials. 

Predictive biomarkers are defined as biomarkers used to identify patient subgroups more likely to benefit from a certain type of therapy. Treating only those patients within the biomarker-defined subgroups with the associated therapeutics should result in an enhanced probability of efficacy relative to an unselected population. Furthermore, patients who would not be expected to benefit would not be exposed to potentially toxic treatments and could be referred to a treatment more likely to be effective.

In the field of targeted anticancer therapy, where the drug mechanism of action consists in the direct interference with an established oncogenic driver (such as epidermal growth factor (EGF) receptor or human epidermal growth factor receptor 2 (Her2) expression), single analyte biomarkers directly measuring the presence or absence of the drug target on tumour cells have traditionally been used in the clinic to identify the patient subset that would likely benefit from the targeted treatment. The situation is quite different for immuno-oncology (IO) drugs, i.e., agents that act on the host immune system, which ultimately constitutes the “therapy,” i.e., fighting the tumour.

The immune response in cancer reflects a series of carefully regulated events that can be self-propagating (defined by Mellman and Chen as the cancer-immunity cycle [11]). Each step of this cycle requires the coordination of many factors, both stimulatory and inhibitory in nature; this is the reason why the assessment of the cancer–immune process should ideally be addressed holistically rather than in its single elements.

In patients with cancer, the cancer-immunity cycle does not perform optimally. However, the rate-limiting step or steps in any given patient may be different. Since the goal of cancer immunotherapy is to initiate or reiterate a self-sustaining cycle of cancer immunity enabling it to amplify and propagate an anticancer response, the most effective approaches will involve selectively targeting the rate-limiting step or steps in any given patient. Thus, there is a need for biomarkers to identify the roadblocks and the underlying biology at the individual patient level to guide an appropriate therapeutic intervention.

It is now recognized that the cancer-immune interaction is influenced by a complex set of tumour genetic and epigenetic factors, as well as by host genomic and environmental factors, which, acting together, govern the strength and timing of the anticancer response. Because of the complexity of the immune response and tumour biology, single analyte biomarkers are not very informative.

Thanks to the rapid progress in technology, today we can measure the factors affecting the cancer-immune interaction using technology platforms that separately measure the different types of potentially informative analytes (DNA, RNA, and proteins). The current fundamental challenges in immuno-oncology (IO) translational research remain the amount of informative data available from small clinical samples and how to integrate the data easily and in a timely fashion into biologically and clinically actionable information.

## 3. Approved and Candidate Biomarkers for IO Therapies

The development of checkpoint inhibitors has been a landmark accomplishment in harnessing the immune system to reject tumours and has set the stage for powerful new genetic analysis tools to revolutionize immuno-oncology. 

Checkpoint inhibition (i.e., antibodies directed against pathways involved in adaptive peripheral immune suppression, such as CTLA-4, PD-1 and PD-L1) is an especially promising anti-tumour strategy that appears to be clinically relevant across a number of tumour types. Furthermore, the generation of memory T cell responses can provide long-term immunity—and this has been borne out by the extended responses observed in the clinic. In addition, on the basis of the improved understanding of the biologic mechanisms underlying cancer immune interactions, a wave of novel immunotherapeutic approaches that target activating and inhibitory T cell receptors, adoptive cell therapies (ACT) including tissue-infiltrating lymphocytes (TILs), chimeric antigen receptors (CARs), T cell receptor (TCR)-modified T cells, and bispecific antibodies are now clinically evaluated, and two chimeric antigen receptor T cell (CAR-T) products have recently been approved in the United States for the treatment of subsets of patients with refractory hematologic malignancies (specifically acute lymphoblastic leukemia and diffuse large B cell lymphoma) [12,13,14].

The following sections review the biomarkers currently in use and under investigation for use with checkpoint inhibitors—primarily those targeting PD-1 and PD-L1, since clinically useful biomarkers to predict the response to anti-CTLA4 treatment remain an unmet need. In addition, different biomarkers targeting different cell populations within a tumour are also being investigated as IO biomarkers for other immunotherapeutic approaches but are at an earlier stage of development, e.g., regulatory T cells (Tregs), lymphocyte activation gene 3 9LAG-3), myeloid-derived suppressor cells (MDSCs), and indoleamine 2, 3-dioxygenase (IDO). Time will tell whether these molecules will play a role in the clinic.

Finally, the efforts to develop predictive biomarkers for use with cancer vaccines and CAR-T therapy are also ongoing but preliminary; biomarkers characterizing the pretreatment immunological status are showing promise in this regard, and in several cases gene signatures have been identified that appear to be useful indicators of an immune status favorable to response [15,16].

## 4. Measuring the Target by Immunohistochemistry (IHC)

The signaling axis consisting of the receptor PD-1 and its ligands PD-L1 and PD-L2 is a negative regulator of T cell function. The role of PD-1 is to dampen an ongoing immune response in the periphery with the objective of controlling tissue damage after infection and inflammation. Currently, multiple antibodies against either PD-1 or PD-L1 are approved, mostly for the treatment of advanced metastatic cancers including metastatic melanoma, metastatic non-small-cell lung cancer (NSCLC), recurrent or metastatic head and neck cancer, refractory classical Hodgkins lymphoma, urothelial carcinoma, gastric cancer, and cancers with a biomarker referred to as high microsatellite instability (MSI-H). For these therapies (with the exception of the latter), PD-L1 IHC has been the primary diagnostic evaluated thus far. Unfortunately, the practice to date has been to independently develop anti-PD-L1 IHC diagnostic assays using different antibodies, platforms, scoring systems, and cutoffs. As a result, the current matrix of therapeutics and diagnostics represents a complex challenge for testing and decision-making in the clinic.

Beyond the issues with current PD-L1 IHC assays, PD-L1 as a single-analyte biomarker has disadvantages: mainly cellular, spatial, and temporal heterogeneity, all of which contribute to the poor prediction accuracy (in particular the poor negative predictive value) of this biomarker in the clinic [17]. Furthermore, the relationship of PD-L1 expression to prognosis is controversial and differs between tumour types [18]. 

Finally, fewer than a quarter to half of patients, even in highly selected cohorts, have experienced a clinical benefit from anti-PD-1 or anti-PD-L1 therapies. A more accurate prediction of response is likely dependent on a more informative measurement of the complex and evolving tumour immune microenvironment and will likely involve more than a single analyte.

## 5. Measuring Tumour Antigenicity: Tumour Mutation Load and Microsatellite Instability (Mismatch Repair Deficiency)

A biomarker that has been associated with response to immunotherapy in multiple cancer types is tumour mutation load. An association between mutation load and response to immune checkpoint inhibitors (CTLA-4 blocking agents in this case) was first observed in studies of ipilimumab in advanced melanoma [19,20], raising the possibility that the genetic landscape of a tumour may affect the clinical benefit provided by immunotherapies.

Tumour mutation load is a measure of the number of mutations within a tumour genome, defined as the total number of mutations per coding area of a tumour genome. There is large variability in mutation load within tumour types, ranging from just a few to thousands of mutations.

Lower-grade and paediatric malignancies tend to have the lowest mutation load, while epithelial cancers associated with environmental DNA damage are most highly mutated. For example, in patients with NSCLC, the tumours in those who have never smoked have fewer somatic mutations compared with the tumours in smokers, which may have 10-fold more mutations [20].

A high tumour mutation load has been shown to be associated with better response rates to checkpoint inhibitors in melanoma [19,21], NSCLC [22,23], and urothelial carcinoma [24]. Lower tumour mutation loads are observed in tumour types exhibiting limited responses to anti-PD-1 and anti-PD-L1 agents, such as colorectal, ovarian, and prostate tumours [25].

Tumour mutation load can be determined by whole-exome sequencing, but widespread access to this method is limited in a clinical setting because of its high cost and bioinformatics requirements; thus, efforts are underway to develop methods to accurately estimate total mutation load from widely available next-generation sequencing gene panels. It has been shown that the mutation load of the whole genome can be inferred from sequencing a much smaller panel of just a few hundred genes [26,27,28]. The mutation load can be estimated using targeted sequencing panels with similar accuracy to that reported using whole-exome sequencing [27,29]. For example, the Foundation One test (Foundation Medicine, Cambridge, MA, USA) is a validated targeted sequencing approach to characterize mutations in 324 genes known to be mutated in solid tumours. As such, the test was approved by the FDA in December 2017 to detect mutations in tumours from patients previously diagnosed with any type of solid tumour for clinical management purposes, including the selection of appropriate FDA-approved treatments in certain cancer types. As a laboratory-developed test, the Foundation One assay has also been used to demonstrate that the mutation load predicts the response to various checkpoint inhibitors in urothelial carcinoma [30], melanoma [31], lung cancer [32], and colorectal cancer [33], and for this indication it is being evaluated prospectively in clinical trials. 

A high tumour mutation load is also associated with mutations in genes for DNA mismatch repair pathways (melanocyte-stimulating hormone (MSH)2, MSH6, MutL homolog 1 (MLH1), post meiotic segregation increased 2 protein (PMS2)), microsatellite instability (MSI), and DNA polymerases (POLE) [29]. Further supporting the association between a high mutation load and the response to immunotherapy, mismatch repair (MMR)-deficient tumours, the genomes of which contain high numbers of somatic mutations, are susceptible to immune checkpoint blockade [34].

Tumour mutation load, measured by comprehensive genomic profiling, is an important emerging biomarker that shows promise in its ability to predict the response to immune checkpoint inhibitors. Further studies are required to fully understand how this novel biomarker can complement the current targeted immunotherapy landscape and its use across multiple tumour types. A multicomponent predictive biomarker system that combines tumour mutation load with other parameters, such as gene and protein expression, neoantigens, MSI status, and immune targets, is likely required to enable physicians to more accurately select patients who will benefit from these therapies.

In 2017, pembrolizumab was approved for the treatment of patients with unresectable or metastatic solid tumours that have been identified as having MSI-H or mismatch repair deficiency (dMMR), thus becoming the first cancer treatment approved by the United States Food and Drug Administration (FDA) on the basis of a common biomarker rather than the tumour cell of origin. According to the new FDA labeling, the presence of dMMR is a sufficient indication for the use of the checkpoint inhibitor pembrolizumab in any unresectable or metastatic solid tumours in an adult or child. The approval was not accompanied by the simultaneous approval of a complementary or companion diagnostic, since laboratory-developed tests measuring dMMP or MSI-H have been available to routinely measure these genetic alterations as part of a genetic syndrome associated with familiar colorectal cancer. 

## 6. Quantification and Characterization of T cells

The potential importance of PD-L1 expression by infiltrating immune cells [17], the presence and location of CD8+ tumour-infiltrating lymphocytes [35], and other factors in the tumour immune microenvironment [36] are currently being studied to discern more sensitive and specific predictors of clinical outcomes. In many tumour types, the presence of tumour-infiltrating lymphocytes is associated with an improved prognosis relative to tumours without an immune infiltrate. The Immunoscore^®^ assay for Colon Cancer (IS Colon; HalioDx SAS, Paris, France), an IHC-based test, was developed in the context of colon tumours, which are known to be immunogenic, and assesses the host immune response by measuring intra- and peritumoural T cell infiltration in formalin-fixed paraffin-embedded (FFPE) tissue sections. The scoring system is derived from the immune contexture (the type, functional orientation, density, and location of adaptive immune cells within distinct tumour regions) and is based on the measurement of two lymphocyte populations (CD3, CD8 or CD8, CD45RO), both in the core and in the invasive margin of the tumours. The Immunoscore has been shown to be a clinically useful prognostic marker in colorectal cancer [37] and is being studied in other tumours as a predictor of the response to checkpoint inhibition. 

In addition to enumerating the T cells within a tumour, it may also be informative to characterize the clonality of the T cell population as a way to infer the expansion of tumour-reactive T cell clones. Immunosequencing is a technology that enables the profiling of T cell and B cell repertoires. Rearranged CDR3 sequences are unique for a given T cell clone and are increased in prevalence as that clone expands in response to antigenic stimulation. The immunosequencing assay captures both specific individual clones as well as the full CDR3 repertoire. The technology is available as a commercial assay, ImmunoSeq (Adaptive Biotechnologies, Seattle, WA, USA), although its clinical utility in this setting has not yet been established. 

ImmunoSeq has been used to sequence the T cell repertoire within tumours of patients with metastatic melanoma after treatment with pembrolizumab to detect differences in the resultant immune repertoire between responders and non-responders [37]. The pretreatment samples from the responders showed a higher proportion of tumour-infiltrating lymphocytes (TILs) and more clonality, while the samples from the non-responders showed lower levels of TILs and greater diversity.

## 7. Gene Expression Signatures

As opposed to the mutated genes in tumours, which remain largely constant, the immune response is dynamic and changes rapidly. Therefore, the issue facing the field of cancer immunotherapy is how to measure an evolving immune response, recognize the immune response that contributes to a clinical benefit, and drive every patient’s immune response in that direction through combination therapies.

Gene expression signatures are potentially the richest source of diagnostic information. A gene expression signature is a combined expression pattern of a group of genes that provides information in terms of diagnosis, prognosis, or prediction of the therapeutic response. 

In immuno-oncology, using gene expression profiling signatures, two major subsets of advanced solid tumours can be identified:

- Approximately one-third of tumours have a T cell-inflamed tumour microenvironment signature (T cell markers, chemokines, macrophage-activated antigens, type I interferon (IFN) transcriptional profile) revealing a pre-existing adaptive immune response and thus suggesting that the expression of local inhibitory factors is at play; for this type of tumours, agents that help to brake the peripheral tolerance are more likely to be clinically successful;
- Non-T-cell-inflamed tumours have no T cell infiltration, suggesting a lack of innate immune activation or a block in T cell trafficking. These tumours require therapeutic approaches that activate the innate immune response by overcoming the central tolerance or by manipulating the oncogene signaling pathways interfering with T cell trafficking.

Currently, there are no approved assays to measure the level of tumour inflammation, although all major companies developing checkpoint inhibitors are working with different research-grade assays [38]. One gene signature, the Tumour Inflammation Signature (TIS), has been developed as a clinical-grade assay that provides both quantitative and qualitative information about the immune environment within a tumour, reporting on the presence of an immune infiltrate as well as the functional status of T cells. The TIS, developed on the NanoString nCounter^®^ gene expression system (NanoString Technologies, Inc., Seattle, WA, USA), is an 18-gene signature that measures peripherally suppressed adaptive immune response within the tumour [2]. The TIS contains IFN-γ-responsive genes related to antigen presentation, chemokine expression, cytotoxic activity, and adaptive immune resistance. 

The TIS was developed by Merck as a clinical-grade trial assay to predict immune response to pembrolizumab [2]. Through a series of training exercises using samples from clinical trials initially in melanoma and then extending to other tumour types, the assay was developed and evolved to define a pan-tumour T cell-inflamed phenotype in 10 cancers (melanoma, bladder, gastric, head and neck squamous cell carcinoma (HNSCC), triple-negative breast cancer, anal canal, biliary, colorectal, esophageal, and ovarian cancer). Subsequent studies have demonstrated the value of the TIS in other clinical settings, such as in melanomas treated with the ipilimumab plus nivolumab in the neoadjuvant setting [39], as well as in melanomas treated with ipilimumab-high dose interferon [40]. In HNSCC tumours, TIS has been shown to have greater sensitivity and improved negative predictive value relative to PD-L1 IHC to detect responders to pembrolizumab. Furthermore, in HNSCC, the mutation load and TIS score were both independently predictive of the response to pembrolizumab in patients negative for human papillomavirus (HPV) and Epstein-Barr virus (EBV); however, only the TIS score was predictive in HPV-positive or EBV-positive patients, in whom, presumably, the viral oncogenes are driving tumorigenesis and the immune response, and the mutation load is lower [41]. 

Traditional methods of gene expression analysis have limitations for clinical applications. For example, reverse transcription polymerase chain reaction (RT-PCR) measures the expression of one gene at a time, whereas multiplex expression profiling techniques such as microarrays, covering many thousands of transcripts, are often expensive and lack flexibility and reproducibility when evaluating low-quality RNA samples such as those from FFPE. Platforms that enable the multiplexed analysis of biomarkers from limited amounts of poor-quality material are therefore very attractive.

The Nanostring Technologies nCounter platform (NanoString Inc., Seattle, WA, USA) is a relatively new technology and has been used within various clinical and research applications. The automated nCounter platform hybridizes fluorescent barcodes directly to specific nucleic acid sequences, allowing for the nonamplified measurement of up to 800 targets within one sample [42]. The TIS, discussed above, was developed on the NanoString platform for use with pembrolizumab.

**Figure 1:**
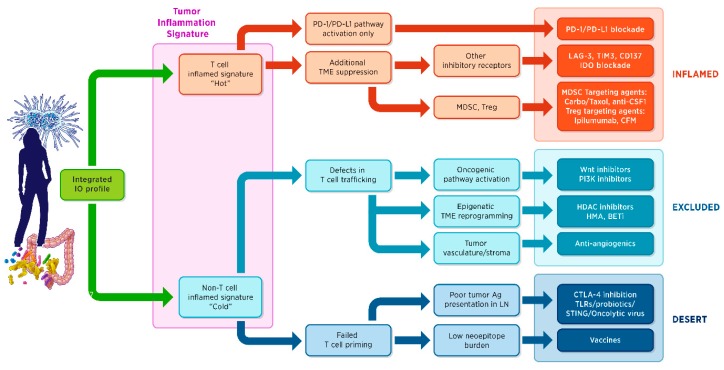
Actionable immune-based classification of cancer. Ag = antigen; BETi = inhibitors of bromodomain and extraterminal proteins; carbo = carboplatin; CSF1 = colony stimulating factor 1; CFM = cyclophosphamide; CTLA-4 = cytotoxic T-lymphocyte–associated antigen 4; HDAC = histone deacetylase; HMA = hypomethylating agents; IDO = indoleamine 2,3-dioxyenase; IO = immune-oncology; LN = lymph nodes; LAG-3 = lymphocyte-activation gene 3; MDSC = myeloid-derived suppressor cells; P13K = phosphoinositide 3-kinase; PD-1 = programmed cell death-1; PD-L1 = programmed cell death-ligand 1; STING = stimulator of interferon genes; TIM3 = T cell immunoglobulin and mucin domain 3; TME = tumor microenvironment; Treg = regulatory T cells; TLR = toll-like receptor; Wnt = wingless, int-1.

As gene expression signatures become more sophisticated, they yield more information that can be used for diagnosis, prognosis, or prediction of the therapeutic response. These powerful assays are harnessing the tremendous diversity and flexibility of the immune system and hold significant promise for the personalized treatment of cancer. Figure 1 proposes a framework for organizing the biologic information to be measured and integrated, ideally in a single assay, to inform an effective drug development and eventually a clinical implementation. Briefly, anticancer immunity in humans can be histologically segregated into three main phenotypes: the inflamed phenotype (also known as “hot”), the immune-excluded phenotype, and the immune-desert phenotype (the latter two considered “cold” tumours) [43]. Importantly, each is associated with specific underlying biological mechanisms that may prevent the host immune response from eradicating the cancer. Identifying these mechanisms at the level of the individual patient is therefore critical for both the development and the clinical implementation of current and new therapeutic approaches. 

The TIS gene expression profiling algorithm described by Ayers et al. (2017) [2] is at the base of this decision tree. However, although necessary, the presence of an adaptive immune response is not always sufficient for a response to the PD-1–PD-L1 blockade. Additional mechanisms of peripheral immune suppression may exist, including other checkpoint inhibitors as well as negative regulatory cell subtypes, e.g., regulatory T cells (Tregs) and myeloid-derived suppressor cells (MDSCs). In the case of the non-inflamed phenotype, the next important question to be answered is whether there are defects in T cell trafficking or in appropriate T cell priming and activation. These may be intrinsic to the tumours (activation of oncogenic pathways that alter the local chemokine state; presence of vascular factors, barriers, or stromal-specific inhibition)—or specific to the host. 

The PanCancer IO 360™ assay (NanoString Technologies Inc., Seattle, WA, USA) is a research panel for gene expression measurement designed in the context of the conceptual framework described in Figure 1 to evaluate variables relevant to mechanisms of immune evasion that can potentially be modulated through therapeutic intervention. Using a single sample, a single assay profiles the tumour-immune stroma interactions using a 770-gene expression panel and an associated suite of analysis tools and services. The IO360 panel is designed to be used with solid tumour tissues (excisional biopsies, core needle biopsies, or resected material) and is compatible with FFPE, fresh or frozen tissues, or purified RNA.

The panel encompasses approximately 13 different biological processes and 46 prototype signatures including the TIS and allows for the parallel assessment of additional mechanisms of immune evasion operating in the context of an inflamed tumour phenotype (such as additional checkpoints inhibitors and/or suppressive immune cell populations or immune metabolites) and also in the context of an “immune-excluded” or “immune-desert” tumour phenotype (such as the activation of an oncogenic pathway affecting immune cell trafficking or the intrinsic alteration of the antigen presentation process). Key biological activities that shape the immune response to the tumour are measured, including dMMR, antigen presentation, tumour cell proliferation, cytotoxic activity, glycolysis, and oncogenic pathways. These biological activities have been captured as multigene signatures.

The IO360 panel supports the development of signatures to potentially predict a patient response to a variety of immunotherapeutic interventions. Within the framework of the panel, the biology of the tumour can be matched with the mechanism of action of a particular drug. The content of the panel was selected to be informative across multiple tumour types, and the gene signatures that are included profile mechanisms of immune evasion that are utilized by a wide variety of tumours; thus, it is anticipated to enable a rapid development of novel signatures to predict a tumour response to immunotherapy.

## 8. Challenges and Future Directions in the Clinical Development of IO Biomarkers 

With new omics technologies that provide a much richer profile of the dynamic immune tumour microenvironment, the fundamental challenge in immune-oncology translational research is no longer what target to study, but how much informative data can be produced from one single and precious clinical sample and how to easily integrate the data into biologically and clinically actionable information in the context of a fast-changing therapeutic landscape. Specifically, beyond PD-1–PD-L1 and CTLA-4, other checkpoint inhibitors are being developed, as well as new IO approaches, such as cancer vaccines, adoptive cell therapy with chimeric antibody receptor-expressing T cells (CAR-T), small molecule modulators of immune response (e.g., toll-like receptor (TLR) agonists), and nucleic acid-based therapies. As they reach the clinic, these treatments will likely require new diagnostics to select patients, and biomarkers to monitor the immune responses. Competing groups will need to work together to develop common assays focused on disease biology and therefore useful across multiple drugs sharing the same mechanism of action. Regulatory agencies will likely demand greater efficacy (i.e., more accurate definition of subpopulations likely to benefit), further contributing to the need for accurate and precise predictive tests. 

By analyzing hundreds or thousands of genes simultaneously, next-generation technologies provide vast amounts of data that permit the identification of actionable mutations to guide the therapeutic decision-making. Assays such as the IO360 pan-cancer tool measure and integrate multiple signals on the basis of the biology of a given tumour and of the host immune system. Ideally, a “universal test” would provide a defined set of treatment options across all therapeutic modalities (immune-oncology, targeted therapy, chemotherapy) as well as the benefit–risk profile of a given drug for a given patient.
